# Analysis of the life course effects of the disability dilemma among rural older adults in China

**DOI:** 10.3389/fpubh.2024.1358106

**Published:** 2024-05-27

**Authors:** Fen Li, Xiangdong Gao, Yahui Meng

**Affiliations:** ^1^School of Management, Xuzhou Medical University, Xuzhou, Jiangsu, China; ^2^School of Public Administration, East China Normal University, Shanghai, China; ^3^Xuzhou Cancer Hospital, Xuzhou, Jiangsu, China

**Keywords:** healthy aging, rural older adults in China, disability dilemma, life course, prevention

## Abstract

**Objective:**

To analyse whether the accumulation of early adverse experiences among individuals of different generations has an impact on disabilities and evaluate the cumulative effects of disadvantages in rural older adults in China.

**Methods:**

A Binary Logit Model was used to analyse the life course effects of the disability dilemma among rural older adults.

**Results:**

Regarding Activities of Daily Life (ADLs), there was no significant difference between older adults that experienced 1 adverse events and the control group. The probability of older adults experiencing difficulties in 2, 3, 4, or more types of ADLs was 1.486 times, 2.173 times, and 3.048 times higher than that of the control group, respectively. Regarding Instrumental Activities of Daily Life (IADLs), there was no significant difference between the population that experienced 1 or 2 adverse events and the control group. The probability of experiencing difficulties in 3, 4, or more types of IADLs was 1.527 times and 1.937 times higher than that of the control group, respectively. Early adverse events had a cumulative disadvantageous effect on disability in older adults. The longer the duration of adverse experiences, the higher the risk of disability in old age. Education had a significant mitigating effect on health risks.

**Conclusion:**

Pay attention to early factors in the life course, strengthen the promotion of health prevention concepts, and pay attention to the moderating and relieving effects of education on health. We should also gradually improve the rural disability care system and family health security capabilities in China’s rural areas.

## 1 Introduction

Exploring the cumulative effects of disability and disadvantages among rural older adults in China from a life course perspective to achieve healthy aging is inherently necessary and an important way to shift the prevention threshold forwards and intervene in the influencing factors of health (1–5). Promoting healthy aging and ensuring the health rights of older adults are important parts of helping China Modernization. According to the Seventh Population Census Data of China, in rural areas, the population is shrinking, and the proportion of the rural population to the total population is 36.11%. Compared with the Sixth Population Census of China, the rural population has decreased by 164.4 million, while the proportion of older adults aged 60 and above living in rural areas is 7.99 percentage points higher than that living in urban areas (6–8). With the gradual increase in population migration and mobility in rural areas of China, the transformation of the family structure, and the continuous advancement of the aging process, the demand for healthy aging of the older adult population has also increased. Health is an important cornerstone of individual survival and development, and disability has become one of the important factors affecting the healthy aging of rural older adults (9, 10). Over time, do the early experiences of individuals born in different eras have an impact on disability in old age? Do these experiences have a cumulative disadvantageous effect on disabilities in older adults? This is the key issue discussed in this article.

## 2 Materials and methods

### 2.1 Data sources

The data used in this article came from the China Health and Retirement Longitudinal Survey (CHARLS) from 2014 and 2018. CHARLS 2014 is the “Life Course Special Survey,” and CHARLS 2018 is the fourth follow-up survey. This article used the individual’s ID as the identification condition to merge data from the 2014 and 2018 surveys. Based on research needs, we performed logical matching, merging, filtering, and cleaning of the data to obtain the required variables. With both registered residence and residence in rural areas as the criteria, 5,017 older adults were selected as the research objects.

### 2.2 Variable selection and feature description

#### 2.2.1 Disability indicators and descriptions

This study used self-care ability to measure the disability status of older adults, including whether they had difficulties in ADLs, IADLs, and their subitems. In the CHARLS questionnaire, ADL and IADL scales were used to measure the disability status of rural older adults (11, 12). In the questionnaire, the independently completed ADL and IADL scales are divided into four levels: 1 = no difficulty, 2 = difficulty but still achievable, 3 = difficulty requiring help, and 4 = inability to complete. For the purpose of this research, the disability status of older adults was divided into binary variables (13). If there were no difficulties in any of the subitems in the ADL scale for rural older adults, then ADL = 0 was defined; if there were difficulties in at least one ADL, ADL = 1 was defined. Similarly, when rural older adults had no difficulties in any IADLs, then IADL was defined as 0; when they had difficulties in at least one IADL, IADL = 1.

From ADLs, such as bathing (10.91%), getting into/out of bed (10.79%), and Getting up from/off the toilet (19.15%), had a higher proportion of difficulties. However, the proportion of activities such as dressing (8.80%), eating (3.77%), and controlling urination and defecation (6.45%) that were small in scope and intensity and essential for maintaining a regular life had a smaller proportion of difficulties. The proportion of people who had difficulties in IADLs, especially the ability to perform household chores (18.40%), cooking (13.50%), shopping (12.28%), making phone calls (27.78%), Taking medications (6.38%) and managing finances (14.32%) were greater than that of people who had difficulties in ADLs. In addition, the proportion of difficulties in at least one ADL was 28.21%, and the proportion of difficulties in at least one IADL was 43.84%. Therefore, for rural older adult individuals, the proportion of IADL difficulties was higher than that of ADL difficulties.

#### 2.2.2 Main characteristics and descriptions

When exploring the reasons for the disability dilemma of older adults in rural China, based on life course theory, the individual characteristics selected were gender, age, and education. The early variables in the life course were health status before age 15, duration of early adverse experiences, number of early adverse events, adult health status, and occupation in adulthood. The characteristics of older adult individuals were body mass index, self-assessment of memory, measurement of mental health, self-assessment of mental health, medical insurance, pension insurance, duration of sleep at night, and whether they nap, smoke, and drank alcohol in the past year.

This study selected 10 types of adverse events from childhood and adolescence, as well as youth and adulthood, for early life experiences. Among them, adverse experiences of children and adolescents included biological parents divorced before the age of 17, a bedridden father or mother, a father or mother with mental impairments, a father or mother with serious disabilities, a father or mother who passed away before the individuals was aged 17, being bullied by neighboring children when young, being detained before the age of 17, and family experience of hunger before the age of 17. Adverse experiences in youth and adulthood included whether the individual experienced serious physical injuries after the age of 16 and whether they ever left work for more than a month due to physical health reasons. First, the 10 selected adverse events or experience indicators were summed to obtain the “number of adverse events” indicator. The value range of the indicators was 0–10, and the larger the value, the more adverse events experienced. Second, we examined the duration of adverse events: for the 10 adverse events selected in this article, the duration of the experience of hunger was a convenient indicator for calculating the duration of adverse events. Therefore, we used the period and duration of hunger as proxy variables to represent the duration of adverse events. The assignment and characteristics of the variables are shown in Table 1.

**Table 1:** Assignment and description of variables.

Variables	Assignment	Observations	Mean	Standard deviation
ADLs	0 = No difficulty, 1 = Difficulty	5,002	0.282	0.450
IADLs	0 = No difficulty, 1 = Difficulty	4,952	0.438	0.496
Gender	0 = male, 1 = female	5,017	0.52	0.50
Age	1 = 60–64,2 = 65–69,3 = 70–74,4 = 75–79,5 = 80 years old and above	5,015	67.55	6.57
Education	1 = Uneducated, 2 = Primary school and below, 3 = Junior high school and above	4,761	1.74	0.69
Health status before 15 years of age	0 = poor, 1 = good	4,976	0.14	0.34
Duration of early adverse experiences	0 = none, 1 = 1 stage, 2 = 2 stages, 3 = 3 stages	4,884	1.46	1.08
Number of early adverse experiences	0–10	5,017	1.85	1.19
Health status during adulthood	1 = poor, 0 = good	4,994	0.18	0.38
Occupation type during adulthood	1 = Agriculture, 2 = Non-agriculture, 3 = Agriculture+non-agriculture, 4 = None	4,967	–	–
BMI	0 = normal, 1 = abnormal	5,017	0.537	0.499
Memory self-assessment	0 = good, 1 = poor	4,973	4.267	0.794
Measurement of mental health	0 = good, 1 = poor	5,017	0.414	0.493
Self-evaluation of mental health	0 = good, 1 = poor	4,908	0.083	0.275
Medical insurance	0 = not participating, 1 = participating	4,767	0.933	0.250
Pension insurance	0 = not participating, 1 = participating	5,017	0.815	0.388
Duration of sleep at night	1 = less than 4 h, 2 = greater than 4 but less than 6 h, 3 = greater than 6 but less than 8 h, 4 = greater than 8 h	4,847	2.383	0.959
Napping	0 = no, 1 = yes	4,948	0.572	0.495
Smoking	0 = no, 1 = yes	4,854	0.473	0.499
Alcohol consumption	0 = no, 1 = yes	5,013	0.317	0.465

Due to varying degrees of missing variables, the number of observations varies. The occupation type during adulthood was a non-hierarchical variable, so the mean and standard deviation were not calculated.

### 2.3 Method and model construction

To evaluate the disability dilemma among older adults in rural China, it was first necessary to construct a basic equation for the impact of early life process factors on older adult disability status as shown in equation (1):


(1) $$H_{i}=\alpha_{0}+\alpha_{1}X_{i}+\alpha_{2}X_{j}+\varepsilon$$

Where $H_{i}$ is the health status of individual i in the older adult stage, $X_{i}$ is a certain life process variable, $X_{j}$ is other variables controlled for when analysing the impact of $X_{i}$ on the current health status, and ε is a random perturbation term. When performing analyses based on this basic equation, the indicators of ADLs and IADLs were treated as binary variables, with 0 indicating no difficulties and 1 indicating difficulties. A binary logit model was used to analyse the causes of the disability dilemma among rural older adults.

The Logit Model and form were as follows: $f\left(p\right)=\alpha +\beta X+\varepsilon$ . To analyse the factors contributing to the disability dilemma of rural older adults, p represents the probability of difficulty in older adults’ ability to take care of themselves, $p/\left(1-p\right)$ represents the “probability ratio,” and the “probability ratio” represents the probability of difficulty being $p/\left(1-p\right)$ times higher than the probability of difficulty not being present. Here, X represents the explanatory and control variables included in the logit model, and ε represents the error term in the dependent variable that cannot be explained by the dependent variable. Based on equation (1), we constructed a set of logit models as shown in equations (2), (3):


(2) $$y_{ADL}=\beta_{0}^{ADL}+\beta_{1}^{ADL}x_{1}+\beta_{2}^{ADL}x_{2}+\cdots +\beta_{k}^{ADL}x_{k}+\varepsilon^{ADL}$$

(3) $$y_{IADL}=\beta_{0}^{\mathrm{IADL}}+\beta_{1}^{\mathrm{IADL}}x_{1}+\beta_{2}^{\mathrm{IADL}}x_{2}+\cdots +\beta_{k}^{\mathrm{IADL}}x_{k}+\varepsilon^{\mathrm{IADL}}$$

The Biomedical Ethics Review Committee of Peking University approved CHARLS, and all participants were required to provide written informed consent. The ethical approval number was IRB00001052-11015.

## 3 Results

### 3.1 The impact of the number of adverse experiences on the disability dilemma of rural older adults

Based on the model constructed in this article, we analysed the impact of the number of early adverse events on the disability status of older adults in rural China. The results are shown in Table 2. The constant terms were significant, and the fitting degree of the model was good. For ADLs, there was no significant difference in ADLs between the 1 type group and the group who had not experienced any adverse events in the early stage. From the early experiences of groups with difficulties in 2, 3, 4, and more ADLs, the probability of difficulty in ADLs was 1.486 times, 2.173 times, and 3.048 times higher than that of the control group, respectively. For IADLs, there was no significant difference between the population who experienced one or two adverse events in the early stage and the control group. The probability of experiencing difficulties in the early stages in 3, 4, or more ADLs was 1.527 times and 1.937 times higher than that of the control group, respectively. The number of early adverse experiences significantly affected the disability status of older adults. The more early adverse experiences there were, the higher the probability of disability in older age.

**Table 2:** Estimated results of the impact of the number of early adverse experiences on the disability status of older adults in rural China.

Variables	ADLs	IADLs
Number of early adverse events (none)		
1 type	1.132 (0.131)	1.014 (0.104)
2 types	1.486^***^ (0.174)	1.163 (0.123)
3 types	2.173^***^ (0.274)	1.527^***^ (0.178)
4 types or more	3.048^***^ (0.432)	1.937^***^ (0.262)
Gender (male)	1.259^**^ (0.128)	1.511^**^ (0.143)
Age (60–64 years old)		
65–69 years old	1.416^***^ (0.114)	1.324^***^ (0.098)
70–74 years old	1.767^***^ (0.160)	2.025^***^ (0.172)
75–79 years old	2.197^***^ (0.258)	2.815^***^ (0.321)
80 years old and above	2.812^***^ (0.414)	3.762^***^ (0.568)
Education (uneducated)		
Primary school and below	1.038 (0.077)	0.570^***^ (0.039)
Junior high school and above	0.966 (0.113)	0.480^***^ (0.051)
Health before 15 years of age (poor)	0.821^**^ (0.073)	0.775^***^ (0.062)
Health status during adulthood (good)	1.365^***^ (0.111)	1.549^***^ (0.116)
Occupation type during adulthood (agriculture)		
Non-agriculture	0.916 (0.215)	0.494^***^ (0.103)
Agriculture + non-agriculture	0.916 (0.069)	0.679^***^ (0.044)
None	2.048 (1.031)	1.937 (0.262)
BMI (normal)	1.365^***^ (0.112)	1.161^**^ (0.070)
Memory self-assessment (good)	1.943^***^ (0.127)	1.986^***^ (0.121)
Measurement of mental health (good)	1.848^***^ (0.124)	1.764^***^ (0.111)
Self-evaluation of mental health (good)	1.800^***^ (0.193)	2.177^***^ (0.249)
medical insurance (not participating)	0.777^**^ (0.096)	0.883 (0.108)
pension insurance (not participating)	0.956 (0.081)	0.939 (0.075)
Duration of sleep at night (<4 h)		
4–6 h	0.667^***^ (0.057)	0.689^***^ (0.058)
6.1–8 h	0.537^***^ (0.046)	0.694^***^ (0.058)
>8 h	0.569^***^ (0.066)	0.940 (0.101)
Napping (no)	1.233^***^ (0.081)	1.075 (0.065)
smoking (no)	1.083 (0.100)	1.126 (0.098)
Alcohol consumption (no)	0.810^**^ (0.062)	0.819^**^ (0.058)
Constant	0.172^***^ (0.042)	0.525^**^ (0.120)
samples	4,136	4,094
Prob > chi2	0.000	0.000
*R* ^2^	0.125	0.152

The parentheses after the independent variable indicate the control group. The coefficients in the table represent the odds ratio. **p < 0.05, ***p < 0.01.

Health status before the age of 15 significantly affected the disability status of rural older adults. People with good health status before the age of 15 were 0.821 times and 0.775 times more likely to have difficulties with ADLs and IADLs in older age compared to those with a poor early health status, respectively. That is, older adults with good health status before the age of 15 had a relatively low probability of disability. Health status in adulthood significantly affected the ability to take care of oneself in old age. Older adults with poor health status in adulthood were 1.365 times and 1.549 times more likely to have difficulties with ADLs and IADLs compared to those with poor health status in adulthood. Poor health in adulthood led to a relatively high probability of disability in old age. From the perspective of occupation type in adulthood, the impact of occupation type in adulthood on ADLs in older adults was not significant, but it had a significant impact on IADLs. Older adults engaged in non-agricultural or agricultural+ non-agricultural activities also had relatively good IADL abilities.

From the perspective of control variables, as age increased, the likelihood of difficulties in older adults’ ability to take care of themselves increased, and the likelihood of difficulties in IADLs increased with age. There was a significant gender difference in the disability status of older adults, with male older adults having better self-care abilities on average than female older adults. Older adults with poor BMI, poor memory self-evaluation, poor measured mental health, or poor self-evaluated mental health had a higher probability of disability. Participating in medical insurance protected against disability. A sufficient sleep duration and afternoon naps had a positive effect on older adult individuals’ ability to take care of themselves. The impact of pension insurance and smoking on their ADLs was not significant. Older adults who consumed alcohol in the past year had relatively good self-care abilities, which may be due to reverse selection; that is, older adults with poor self-care abilities tend to avoid drinking behavior to a certain extent.

The level of education had a significant impact on the IADLs of older adults in rural China. The results are shown in Table 2, the probability of difficulty in IADLs in primary school and below was 0.570 times higher than that of the control group (uneducated), and the probability of difficulty in IADLs in junior high school and above was 0.480 times that of the control group (uneducated). That is, older adults with high levels of education were relatively less likely to have difficulties with IADLs. Studies have shown that education level, as one of the important indicators of socioeconomic status, presents a significant mediating effect between early adverse experiences and IADLs disability in older adults, the more early adverse experiences there are, the lower the level of education, and the lower the ability to acquire health capital (14). Education level was a factor that was present throughout the entire life course from the early stages. The education level of rural older adults was closely related to their early life background and experiences. The different early life backgrounds and experiences to some extent led to different opportunities and degrees of early education, which affected career choices throughout the life course, further directly or indirectly affecting the opportunities to obtain health resources during the life course, thus causing differences in the disability status of rural older adults to a certain extent.

### 3.2 The impact of duration on the disability dilemma of rural older adults

The estimation results in Table 3 show that with the addition of control variables (the significance and impact effects of the control variables are not listed separately), the constant terms are significant, and the model fits well. The duration of early adverse events had a significant impact on the disability status of rural older adults. The longer the duration of early adverse events, the higher the probability of disability in old age. Taking no adverse events as the control group, the duration of early adverse events for one stage had no significant impact on the disability status of older adults. For ADLs, the probability of experiencing 2 or 3 stages of early adverse events regarding self-rated health was 1.341 times and 1.257 times higher than that of the control group, respectively. For IADLs, the probability of difficulty in early adverse events lasting for 2 and 3 stages was 1.167 and 1.273 times higher than that of the control group, respectively. As early adverse events continued, the growth rate of health capital slowed or the depreciation rate of health capital gradually increased. The continuous impact of adverse events on health accumulate with age, affecting the ability to take care of oneself in old age.

**Table 3:** Estimated results of the impact of early adverse event duration on the disability status of rural older adults.

Variables	ADLs		IADLs	
	1	2	1	2
Early adverse experience duration (none)	1.083^***^ (0.030)		1.087^***^ (0.029)	
1 stage		1.123 (0.095)		1.061 (0.083)
2 stages		1.341^***^ (0.134)		1.167^*^ (0.110)
3 stages		1.257^***^ (0.112)		1.273^***^ (0.108)
control variables	Yes	Yes	Yes	Yes
Constant	0.270^***^ (0.039)	0.204^***^ (0.046)	0.640^***^ (0.084)	0.557^**^ (0.120)
samples	4,071		4,030	
Prob>chi2	0.000		0.000	
*R* ^2^	0.116		0.149	

The parentheses after the independent variable indicate the control group. The coefficients in the table represent the odds ratio. *p < 0.1, **p < 0.05, ***p < 0.01.

The impact of early adverse events on the disability status of rural older adults varied with the number and duration of adverse events, and early disadvantages accumulated in the older adult population. One of the earliest descriptions of risk/disadvantage accumulation was proposed by Riley JC in 1989, who stated that early adverse experiences can cause long-term damage to physical health through gradual accumulation (15, 16). The accumulation of early adverse factors can have an impact on individual health trajectories, allowing people to experience different lifestyles and obtain different health resources, thereby leading to varying levels of self-care ability among older adults to a certain extent. These adverse factors can be referred to as disadvantages in the life course, and their impact on disability in older adults can be independent or interactive, thereby having a cumulative effect on disability in older adults (17, 18). From the life course perspective, individual early experiences and health endowments are transmitted through the accumulation of life course, and to a certain extent, they constrain and affect the self-care ability of older adult individuals, causing them to fall into the disability dilemma (19, 20). However, when older adults reach a certain age, the importance of early adverse factors on their ability to take care of themselves decreases, and the role of intrinsic biological functions gradually becomes prominent. Rural older adults who did not experience adverse events in early life had a longer life expectancy and a lower risk of disability compared to those who experienced more adverse events in early life.

## 4 Conclusion

Based on the above analysis, the following conclusions can be drawn: (1) the disability dilemma of older adults in rural China is a result of the interaction and transmission of factors in old age and early life, and early adverse events increase the risk of disability in older adults through the cumulative effect of disadvantages. (2) The number and duration of early adverse experiences significantly affect the disability status of older adults in rural China. The more and longer the number and duration of early adverse experiences, the higher the probability of disability in old age, and older adults without adverse experiences tend to live longer. Lifestyle and security conditions in old age significantly affect the health status of older adults in rural China. (3) Individuals with higher levels of education have more opportunities to access health resources and the ability to resist health risks during their growth process. To a certain extent, education can regulate and alleviate the cumulative disadvantageous effect of early adverse experiences on disability in old age.

To further improve the health status of older adults in rural China, first, actively the population must be actively encouraged to adopt the concept of “not getting old” to prevent “getting old.” The occurrence of health risks in the early stages of the life course must be prevented, the cumulative effects of health risks must be reduced, and the health status of older adults must be improved. Second, the prevention of “advanced age” will enhance the health awareness of older adults in rural China. The prevention perspective is not only based on the health of the underage population to preserve their health in old age but also actively encourages the improvement of the health status of young people to prevent a poor health status. The concept of prevention promotes the transformation from passively seeking health services after disability to proactively seeking health care without disability and improves people’s awareness of prevention and health care. This transformation can not only effectively improve the overall health status of rural older adults but also save many medical resources. The prevention threshold must be moved forwards, prevention first health policies must be implemented, and healthy aging must be promoted.

## Data availability statement

The original contributions presented in the study are included in the article/supplementary materials, further inquiries can be directed to the corresponding author.

## Ethics statement

The studies involving humans were approved by the Biomedical Ethics Review Committee of Peking University approved CHARLS, and all participants were required to provide written informed consent. The ethical approval number was IRB00001052-11015. The studies were conducted in accordance with the local legislation and institutional requirements. The participants provided their written informed consent to participate in this study.

## Author contributions

FL: Writing – original draft. XG: Writing – review & editing. YM: Writing – review & editing.
